# Early Warning of Infectious Disease Outbreaks Using Social Media and Digital Data: A Scoping Review

**DOI:** 10.3390/ijerph22071104

**Published:** 2025-07-13

**Authors:** Yamil Liscano, Luis A. Anillo Arrieta, John Fernando Montenegro, Diego Prieto-Alvarado, Jorge Ordoñez

**Affiliations:** 1 Grupo de Investigación en Salud Integral (GISI), Departamento Facultad de Salud, Universidad Santiago de Cali, Cali 760035, Colombia; john.montenegro00@usc.edu.co (J.F.M.); diego.prieto00@usc.edu.co (D.P.-A.); jorge.ordonez01@usc.com.co (J.O.); 2 School of Basic Sciences, Technology, and Engineering, Universidad Nacional Abierta y a Distancia–UNAD, Barranquilla 080005, Colombia; luis.anillo@unad.edu.co; 3 Department of Public Health, Division of Health Sciences, Universidad del Norte, Barranquilla 080005, Colombia

**Keywords:** disease outbreaks, epidemiological surveillance, social media, infodemiology, artificial intelligence, time series analysis

## Abstract

**Background and Aim:** Digital surveillance, which utilizes data from social media, search engines, and other online platforms, has emerged as an innovative approach for the early detection of infectious disease outbreaks. This scoping review aimed to systematically map and characterize the methodologies, performance metrics, and limitations of digital surveillance tools compared to traditional epidemiological monitoring. **Methods**: A scoping review was conducted in accordance with the Joanna Briggs Institute and PRISMA-SCR guidelines. Scientific databases including PubMed, Scopus, and Web of Science were searched, incorporating both empirical studies and systematic reviews without language restrictions. Key elements analyzed included digital sources, analytical algorithms, accuracy metrics, and validation against official surveillance data. **Results**: The reviewed studies demonstrate that digital surveillance can provide significant lead times (from days to several weeks) compared to traditional systems. While performance varies by platform and disease, many models showed strong correlations (r > 0.8) with official case data and achieved low predictive errors, particularly for influenza and COVID-19. Google Trends and X (formerly Twitter) emerged as the most frequently used sources, often analyzed using supervised regression, Bayesian models, and ARIMA techniques. **Conclusions**: While digital surveillance shows strong predictive capabilities, it faces challenges related to data quality and representativeness. Key recommendations include the development of standardized reporting guidelines to improve comparability across studies, the use of statistical techniques like stratification and model weighting to mitigate demographic biases, and leveraging advanced artificial intelligence to differentiate genuine health signals from media-driven noise. These steps are crucial for enhancing the reliability and equity of digital epidemiological monitoring.

## 1. Introduction

Emerging infectious diseases pose an increasing threat to both biodiversity and human societies, significantly impacting ecosystems and global public health. Traditionally, epidemiological surveillance has relied mainly on official systems that can sometimes experience delays in the early detection of outbreaks. In this context, digital surveillance has emerged as a complementary tool that captures early signals in real time using data from unconventional sources [1,2,3,4].

Digital platforms such as search engines, social media, and participatory surveillance systems have become key tools for detecting early signs of epidemic activity. Various studies have demonstrated that signals derived from Baidu searches or X (formerly Twitter) posts, for example, can anticipate the detection of diseases like COVID-19 and influenza by one to three weeks prior to official reports from traditional systems [5,6,7,8].

The integration of multiple digital sources, including Google queries, X messages, and alerts from platforms such as HealthMap, achieves high correlation levels with official data while significantly enhancing predictive capacity. Advanced techniques such as machine learning algorithms, autoregressive models, and temporal smoothing statistical methods have been shown to increase precision and provide timely alerts [4,9,10].

These methods are not confined to a single geographical context; their effectiveness has been validated in multiple countries, including China, the United Kingdom, the United States, and various Latin American nations. The high spatial and temporal resolution of these digital data facilitates the precise identification of local transmission hotspots, enabling targeted prevention and control measures [11,12,13,14,15].

Nevertheless, digital surveillance faces significant challenges related to data quality, such as media noise and biases from disparities in internet access. To ensure the reliability of these signals, rigorous validation and methodological clarity are essential. For instance, “careful keyword selection” is often cited as a requirement, but this can refer to diverse processes ranging from the use of expert-curated clinical terms to empirically validated, algorithm-based selections. While the potential of digital surveillance is clear, the wide heterogeneity in methods, data sources, and validation strategies makes it difficult for policymakers to assess and compare approaches. This creates a critical knowledge gap between academic research and practical implementation, highlighting the need for a systematic synthesis that maps these different methodologies and their limitations [1,16,17,18].

Therefore, the objective of this scoping review is not to definitively evaluate, but to systematically map and characterize the landscape of digital surveillance for infectious diseases. Specifically, we aim to: (1) identify the key data sources, analytical methods, and performance metrics reported in the literature; (2) categorize the different types of early warning mechanisms employed; and (3) synthesize the primary challenges and limitations discussed, including data quality, biases, and the gap between prediction and implementation, in order to provide a clear overview for researchers and decision-makers.

## 2. Materials and Methods

### 2.1. Protocol and Definitions

This study was conducted in accordance with the Joanna Briggs Institute guidelines for scoping reviews and the PRISMA-SCR framework [19,20]. A detailed protocol was developed outlining the eligibility criteria, search strategies, study selection procedures, data extraction, and descriptive analysis, with the aim of mapping and synthesizing the literature on digital disease surveillance.

Traditional Epidemiological Surveillance: The ongoing, systematic collection, analysis, and interpretation of health-related data essential to planning, implementation, and evaluation of public health practice, closely integrated with the timely dissemination of these data to those who need to know [21,22].

Digital Surveillance: The use of digital data, particularly from social media or other internet-based sources, for the purpose of public health surveillance [16].

Participatory Surveillance: An approach for gathering information from the community to monitor health trends, where members of the community are proactively engaged to regularly report on health events. It complements other sources of surveillance information, such as from health care facilities [23].

Warning Signal: For the purpose of this review, a “warning signal” is defined as a statistically significant deviation from a baseline or expected pattern in a digital data stream (e.g., search queries, social media posts) that suggests a potential increase in infectious disease activity processed and identified by an analytical model [6,24,25,26].

### 2.2. Eligibility Criteria

Empirical studies (observational, experimental, and modeling) as well as systematic or scoping reviews that meet the following criteria were included:

- They report methods and results on digital surveillance of infectious diseases (e.g., influenza, COVID-19, RSV, dengue, etc.).
- They describe study design characteristics, digital data sources, detection methods, temporal advantages, accuracy, and correlations with traditional surveillance systems.
- They are published in peer-reviewed journals and available as full-text, without language restrictions.

Studies were excluded based on the following criteria:

- Did not provide empirical data or focus exclusively on theoretical models lacking validation.
- Focused solely on traditional surveillance without integrating digital data.
- Presented unclear or insufficient results for extracting the required information.

### 2.3. PCC (Population, Concept, Context) Question

- Population (P): Studies that involve the use of digital data (e.g., social media, search engines, mobile applications) for disease surveillance in human populations.
- Concept (C): The characteristics, performance, and methods of digital disease surveillance; variables to be extracted include study design, data sources, detection methods, temporal advantages, detection rates, accuracy, among others.
- Context (C): Studies published in peer-reviewed scientific journals addressing digital surveillance of outbreaks or epidemics at local, regional, national, or global levels.

### 2.4. Search Strategy

A systematic and comprehensive search strategy was designed and executed on 24 February 2025, across eight electronic databases: PubMed, Scopus, Web of Science, Springer, SciELO, Science Direct, Google Scholar, and Redalyc. The strategy was constructed using key terms related to the components of the PCC (Population, Concept, Context) question.

No language or date range filters were applied during the database search. The final selection of studies was based on the eligibility criteria detailed in Section 2.2, which were applied by the reviewers during the screening phases.

The specific search strategy used for each database and the results obtained are detailed below:

1. PubMed

Strategy: (((“digital surveillance” [Title/Abstract] OR “infodemiology” [Title/Abstract] OR “infoveillance” [Title/Abstract])) AND ((“infectious diseases” [MeSH Terms] OR “disease outbreaks” [MeSH Terms] OR “epidemics” [Title/Abstract])) AND ((“social media” [MeSH Terms] OR “Google Trends” [Title/Abstract] OR “Twitter” [Title/Abstract] OR “X” [Title/Abstract])) AND ((“machine learning” [MeSH Terms] OR “forecasting” [Title/Abstract] OR “time series analysis” [Title/Abstract]))).

2. Scopus

Strategy: TITLE-ABS-KEY ((“digital surveillance” OR “infodemiology”) AND (“infectious diseases” OR “epidemic”) AND (“social media” OR “Google Trends” OR “Twitter” OR “X”) AND (“machine learning” OR “forecasting”)).

3. Web of Science (WOS)

Strategy: TS = ((“digital surveillance” OR “infodemiology”) AND (“infectious diseases” OR “epidemic”) AND (“social media” OR “Google Trends” OR “Twitter” OR “X”) AND (“machine learning” OR “forecasting”)).

4. Science Direct

Strategy: (“digital surveillance” OR “infodemiology”) AND (“infectious diseases” OR “epidemic”) AND (“social media” OR “Google Trends” OR “Twitter” OR “X”) AND (“machine learning” OR “forecasting”).

5. Google Scholar

Strategy: allintitle: (“digital surveillance” OR “infodemiology” OR “social media”) AND (“infectious diseases” OR “epidemic” OR “outbreak”).

6. Springer

Strategy: (“digital surveillance” OR “infodemiology”) AND (“infectious diseases” OR “epidemic”) AND (“social media” OR “Google”).

7. SciELO

Strategy: (ti:(vigilancia digital OR infodemiología)) AND (ti:(enfermedades infecciosas OR epidemia)).

8. Redalyc

Strategy: (“vigilancia digital” OR “infodemiología”) AND (“enfermedades infecciosas”).

Note: In search strings that include “Twitter OR X,” both terms were used to ensure comprehensive coverage of the literature published both before and after the platform’s rebranding.

### 2.5. Study Selection and Data Extraction

Following the search, all identified records were exported to Zotero (version 6.0; accessed 24 February 2025) for citation management and removal of duplicates. The remaining records were then uploaded to Rayyan AI version (Rayyan Systems Inc.; 125 Cambridgepark Drive, Suite 301, Cambridge, MA 02140, USA; https://www.rayyan.ai/; accessed 24 February 2025), a web application designed to facilitate collaborative screening. Two independent reviewers (Yamil Liscano and Luis Anillo) screened the titles and abstracts of the identified studies to determine their eligibility. Preselected articles were then subjected to full-text review to confirm compliance with the inclusion and exclusion criteria. Discrepancies were resolved by consensus or consultation with a third reviewer.

To achieve the scoping review’s objective of comprehensively mapping the field, a structured form was developed for the detailed extraction of a wide range of variables. This was intended to capture the heterogeneity in study designs, data sources, analytical techniques, and contextual factors. The form captured the following variables:

- Author and Year
- Study Design
- Digital Data Sources
- Comparison Method Employed
- Geographical Scope of the Study
- Techniques or Algorithms Used for Digital Signal Detection
- Temporal Advantage in Detection
- Reported Detection Indicators or Rates
- Measures of Precision and Performance
- Data Collection Period
- Type of Disease or Outbreak
- Specific Digital Platforms and Tools
- Data Preprocessing Methods
- Analytical Algorithms or Techniques
- Statistical and Performance Metrics
- Spatial Resolution and Temporal Granularity
- Integration with Traditional Surveillance Systems
- Keyword Selection Process
- Measurement of Media Impact
- Demographic and Usage Characteristics

Other key methodological characteristics for critical appraisal were assessed.

**Figure 1:**
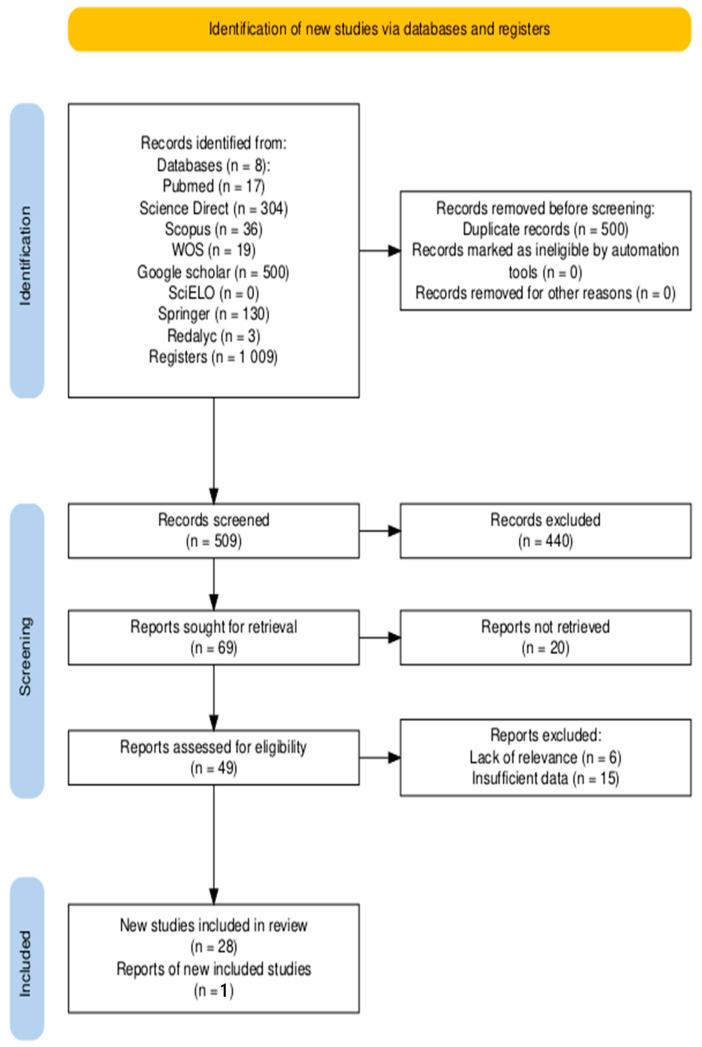
PRISMA flow diagram.

The PRISMA flow diagram summarizing the study selection process is presented in Figure 1. The diagram was generated using the online R package PRISMA2020 [27] (https://estech.shinyapps.io/prisma_flowdiagram/, accessed 24 February 2025).

### 2.6. Statistical Analysis

A descriptive statistical analysis, including frequency counts and distributions of the extracted variables, was conducted using R software (version 4.3.0; accessed 15 March 2025). Foundational charts and plots were generated using R’s ggplot2 library, while more complex and customized visualizations, such as matrices and heatmaps, were created with Python’s matplotlib library (version 3.8.2; accessed 9 April 2025). Napkin (beta version; accessed 6 June 2025; https://www.napkin.ai/) was also used to create schematic and process diagrams.

## 3. Results

### 3.1. General Study Information

A total of 1009 records were identified from the various search sources. After removing 500 duplicates, 509 records remained for screening. Based on title and abstract review, 440 of these were excluded, leaving 69 reports for full-text retrieval. An additional 20 reports could not be retrieved, which left 49 articles to be assessed for eligibility. From these, a further 21 reports were excluded after full-text analysis (15 due to insufficient data and 6 for lack of relevance). This process resulted in 28 studies being included from the database search. Additionally, one eligible study was identified using another method by reviewing the reference lists of included articles. Thus, a total of 29 studies were included in the final review (see Figure 1).

Studies on digital public health surveillance stand out for a remarkable methodological diversity, reflecting an evolution toward more sophisticated approaches. As shown in Table 1, the research includes quantitative comparative empirical analyses (e.g., [5]), observational studies [28], retrospective and cohort analyses [29], as well as exploratory studies [30]. This variety demonstrates how statistical and modeling techniques have progressively adapted to the growing availability of digital data, incorporating advanced methods to improve outbreak prediction and management.

Regarding the diseases studied, respiratory and viral illnesses clearly predominate, especially COVID-19 and influenza, due to their epidemiological significance. The timeframe of data collection is considerable, spanning from early studies on dengue starting in 2003 [46] to recent research focused on COVID-19 [6,28,50]. The urgency of sudden outbreaks like COVID-19 or Zika often leads to studies with limited timeframes, whereas influenza research typically analyzes data across multiple seasons. Beyond the main respiratory viruses, other diseases such as Zika, MRSA, MERS, dengue, and cholera have also been examined. Broad studies like Feldman et al. [42], which covers multiple pathologies, validate the general applicability of these methodologies.

**Figure 2:**
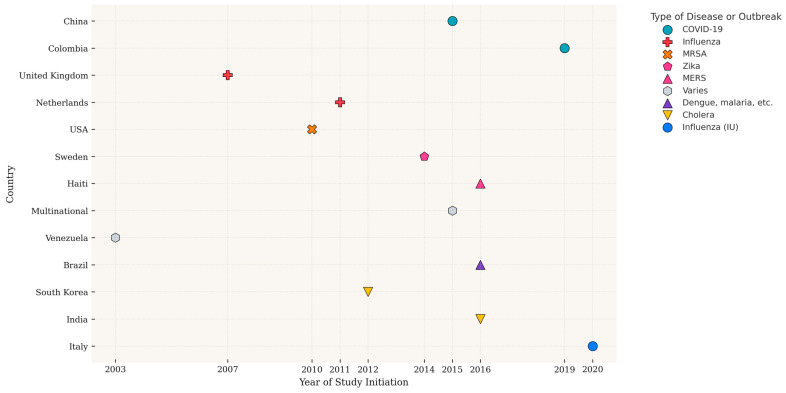
Temporal and geographic distribution of the studies. Note: This figure was generated using the ggplot2 library in R.

Geographical diversity is also a key aspect (see Figure 2). There are studies with a local scope, such as Wittwer et al. [33] in Brazilian cities or Broniatowski et al. [51] in a Baltimore hospital. Other works have national approaches in countries like China, the United Kingdom, the United States, Italy, and India. Additionally, international investigations spanning multiple countries, such as those by Yan et al. [34] and Feldman et al. [42], highlight the scalability of these methods. This geographic breadth allows for the adaptation of methodologies to specific contexts, considering factors like internet connectivity and population density.

### 3.2. Data Sources and Digital Platforms

**Figure 3:**
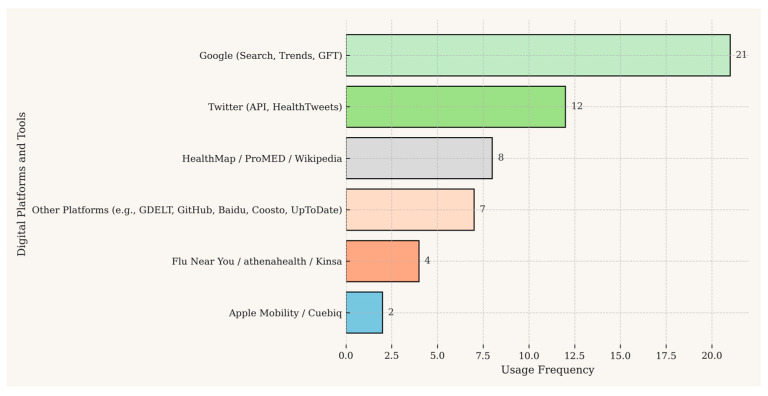
Frequency of use of different data sources. Note: This figure was generated using the matplotlib library in Python.

The analysis of the 29 included studies reveals a digital ecosystem dominated by two primary types of data sources, including public web search engines and social media platforms, as detailed in Table 2 and visually summarized in Figure 3. Search engines, predominantly Google (used in 20 studies), offer high-volume, query-based data that are effective for tracking general interest in diseases like influenza or COVID-19. In contrast, social media platforms like X (12 studies) and Weibo provide richer, albeit noisier, contextual data, often used to analyze symptom self-reporting and public sentiment. A notable trend is the combination of these sources within a single study, a strategy used to balance the breadth of search data with the depth of social media content, as seen in the work of McGough et al. [32] and Santillana et al. [39]

It is important to note that the existing literature is heavily dominated by studies using Google and X. A significant gap exists regarding the use of other globally popular platforms such as Facebook, TikTok, or messaging services like Telegram and WhatsApp, likely due to data access restrictions. This overrepresentation limits the generalizability of the current findings and highlights a key area for future research.

Beyond these, specialized public health platforms such as HealthMap and ProMED-mail serve as crucial aggregators of news and official reports, frequently used to validate signals from other digital sources. Emerging sources are also gaining traction; mobility data from platforms like Apple Mobility and sensor data from smart thermometers were particularly leveraged in COVID-19 studies to correlate population movement and fever trends with case data (see Figure 3).

A near-universal theme across all studies is the validation of digital signals against traditional surveillance systems from institutions like the CDC, WHO, or national health ministries. This pattern underscores that these digital tools are almost exclusively used to complement, by providing early warnings and real-time trends, rather than replace traditional epidemiological reporting.

### 3.3. Methods and Analytical Techniques

**Figure 4:**
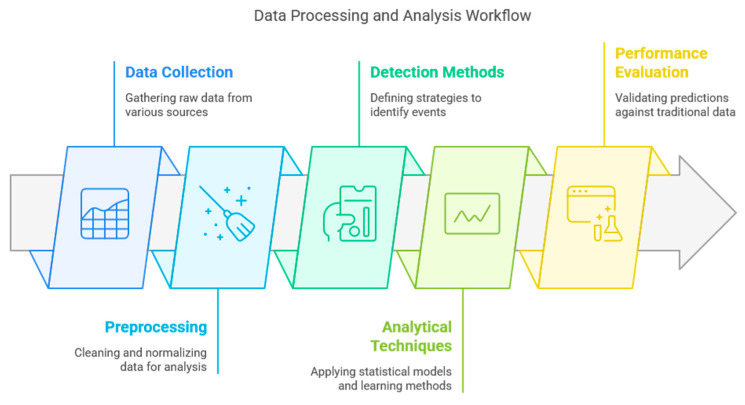
Methodological flow diagram. Note: This figure was generated using Napkin.

The methodological workflow across the reviewed studies, detailed in Table 3, follows a consistent pattern of data collection, preprocessing, signal detection, and analysis, as outlined in Figure 4. In the preprocessing stage, a clear distinction emerges based on the data source. Studies using time series data from search engines frequently apply smoothing techniques like moving averages to reduce noise. In contrast, those analyzing unstructured social media text employ more complex natural language processing techniques, including stop word removal, stemming, and lemmatization to extract meaningful signals.

The methodological flow begins with data collection from a wide array of digital sources, such as search engines, social media, and specialized surveillance platforms, to capture early epidemiological signals (see Figure 4). These raw data then undergo a critical preprocessing phase, where techniques like cleaning, normalization, and smoothing are applied. This process, which often includes content classification to filter out digital noise, is essential for ensuring the quality and reliability of the data before analysis. Subsequently, the analytical stages first focus on detecting predictive patterns and time lags against official reports, using methods such as correlation and causality analysis. Building on this foundation, a varied set of analytical techniques is applied for robust modeling. These range from statistical models like linear regression and ARIMA to advanced machine learning approaches, including supervised regressions, ensemble models, and Bayesian methods, enabling a precise analysis of the captured epidemiological signals.

**Figure 5:**
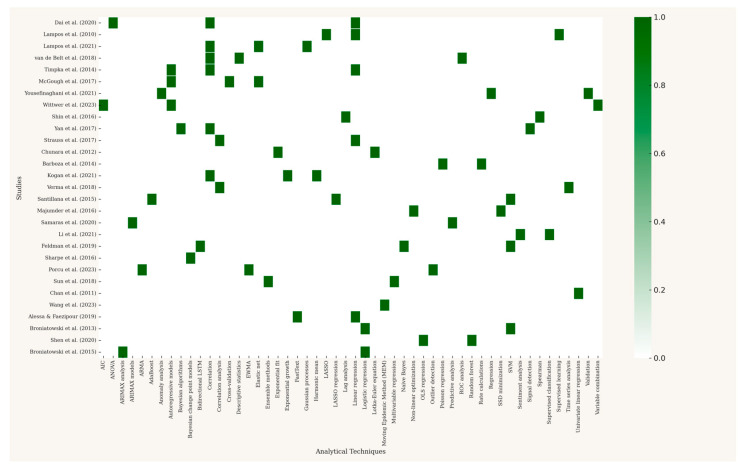
Matrix of analytical techniques in digital surveillance studies (chronologically ordered). Cells marked with a value indicate the presence of a technique in the study, which quickly identifies which methods have been used and how frequently. Note: This figure was generated using the matplotlib library in Python [5,6,8,10,26,28,29,30,31,32,33,34,35,36,37,38,39,40,41,42,43,44,45,46,47,48,49,50,51].

The analytical landscape, visually represented in the matrix in Figure 5, shows an evolution in methodological complexity. While traditional statistical methods like linear regression and correlation analysis remain foundational for validation in many studies [5,29], there is a clear trend toward more sophisticated machine learning techniques. For instance, studies tackling complex, multi-source data often employ supervised regression models like LASSO, ensemble methods like AdaBoost, or advanced classifiers such as support vector machines (SVMs) [39,42]. Similarly, time series forecasting has advanced from standard autoregressive models to more robust ARIMA and ARIMAX models to improve predictive accuracy [10,49].

### 3.4. Performance and Early Detection

The primary value of digital surveillance lies in its performance, specifically its ability to provide early warnings with high accuracy, as detailed in Table 4. The reported lead time varies significantly, from a few days to several weeks ahead of official reports.

**Figure 6:**
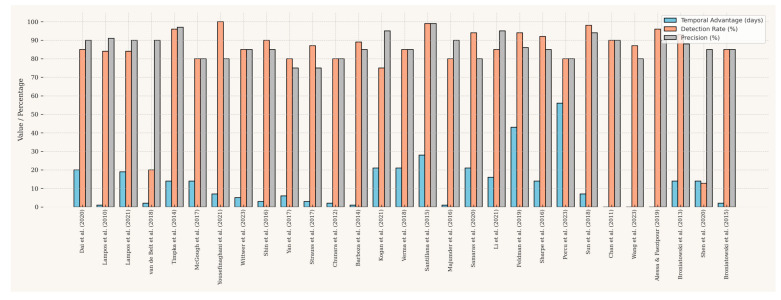
Comparison of lead time and precision across studies. Note: This figure was generated using the ggplot2 library in R [5,6,8,10,26,28,29,30,31,32,33,34,35,36,37,38,39,40,41,42,43,44,45,46,47,48,49,50,51].

While many studies report advantages of 1–3 weeks, exceptional cases exist, such as Feldman et al. [42], who detected outbreaks in news media an average of 43 days before official alerts. However, the practical relevance of such long lead times must be contextualized; for rapidly spreading diseases with short incubation periods like COVID-19, even a few days of early warning can be more impactful for triggering immediate public health responses. A comparative overview of these performance metrics across studies is presented in Figure 6.

**Figure 7:**
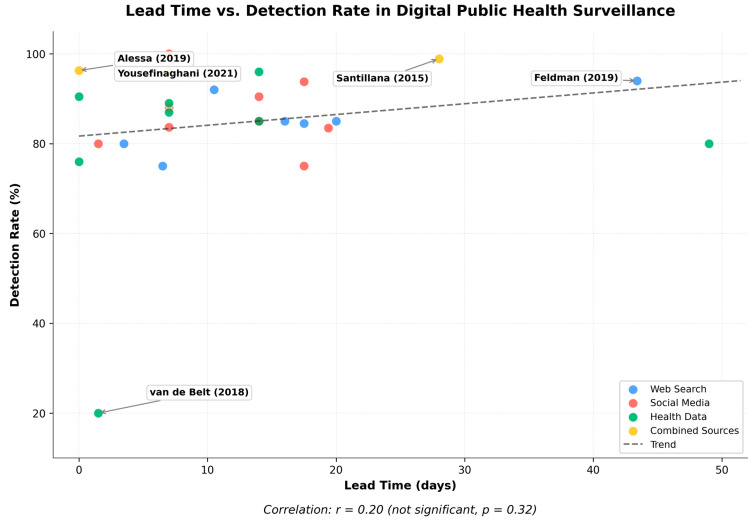
Relationship between lead time and detection rate in digital public health surveillance studies [5,6,8,10,26,28,29,30,31,32,33,34,35,36,37,38,39,40,41,42,43,44,45,46,47,48,49,50,51].

Digital surveillance systems generally demonstrate high precision, with numerous studies reporting strong correlation coefficients (often *r* > 0.8), robust classification metrics (e.g., high sensitivity, specificity, and F1-scores), and low predictive errors (such as RMSE and MAE), as shown in Table 4. However, the trade-off between lead time and precision illustrated in Figure 7 deserves attention. Studies based solely on social media data (red points) sometimes achieve the longest lead times but exhibit considerable variability in precision. In contrast, those leveraging web search data (blue points) tend to maintain consistently high correlation with official reports (*r* > 0.8), though with more moderate lead times. Studies that integrate multiple data sources (yellow points), such as Santillana et al. [39], frequently strike a strategic balance, combining early detection with strong precision. This highlights the potential of multi-source approaches to optimize system performance. Conversely, studies like that of Alessa and Faezipour [48], which achieve high precision but short lead times, are well-suited for nowcasting applications rather than early warning.

The X-axis represents the lead time in days (temporal advantage of digital surveillance methods over official reporting). The Y-axis shows the detection rate as a percentage, reflecting the effectiveness of digital surveillance methods in terms of sensitivity, correlation coefficients, or detection accuracy. Each data point represents a study, color-coded by data source: web search (blue), social media (red), health data (green), and combined sources (yellow). Key studies are labeled for reference. The dashed line indicates the overall trend across all studies (r = 0.20, not significant, *p* = 0.32), suggesting no strong relationship between lead time and detection effectiveness in digital health surveillance systems. Note: This figure was generated using the matplotlib library in Python.

### 3.5. Complementary and Specific Aspects

**Figure 8:**
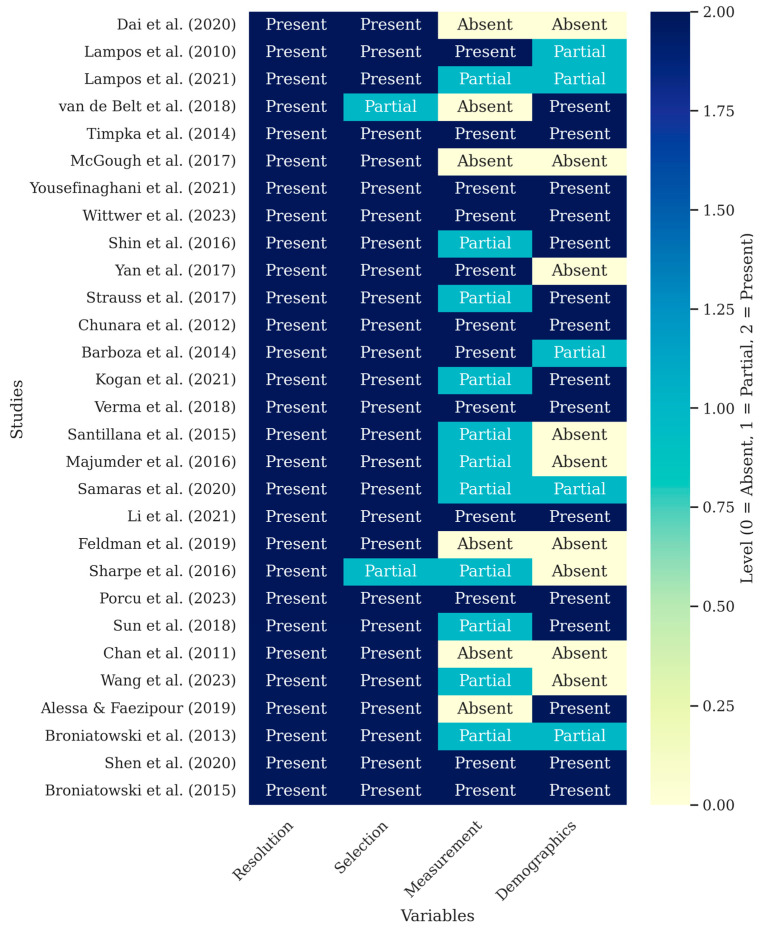
Heat map of complementary characteristics and contextual variables [5,6,8,10,26,28,29,30,31,32,33,34,35,36,37,38,39,40,41,42,43,44,45,46,47,48,49,50,51].

A critical analysis of the studies’ contextual variables, summarized in Table 5 and the Figure 8 heatmap, reveals uneven methodological rigor across the literature. While most studies adequately address spatial and temporal resolution and keyword selection, significant gaps emerge when comparing how they handle external biases.

For instance, regarding spatial resolution, there is a wide range from hyper-local analyses of a single hospital [49] to global surveillance systems [42], a factor largely dictated by data availability. Similarly, the keyword selection process varies from manual, expert-defined lists to automated, correlation-based methods like that used by Verma et al. [38].

In contrast to the detailed reporting on these aspects, the measurement of media impact and the inclusion of demographics represent significant weak points. Only a minority of studies, such as Lampos et al. [28] and Kogan et al. [6], explicitly attempt to model or mitigate the confounding effects of media coverage. Even more sparse is the consideration of demographic biases; few studies outside of those using clinic-level data [29] stratified their analysis by age, gender, or socioeconomic status. This methodological asymmetry, clearly visualized in Figure 8, suggests that while digital surveillance is technically advancing, its findings may be biased by underestimating the human and communication factors that shape digital data.

In this heat map, the level of detail with which each study addresses four fundamental aspects is shown: the spatial and temporal resolution of the data (Resolution), the keyword selection process (Selection), the measurement of media impact (Measurement), and the consideration of demographic or usage variables (Demographics). The color indicates a range from “absent” (0) to “present” (2), allowing an at-a-glance view of each study’s methodological strengths and possible gaps. Note: This figure was generated using the matplotlib library in Python.

## 4. Discussion

### 4.1. Main Findings and Methodological Evolution

**Figure 9:**
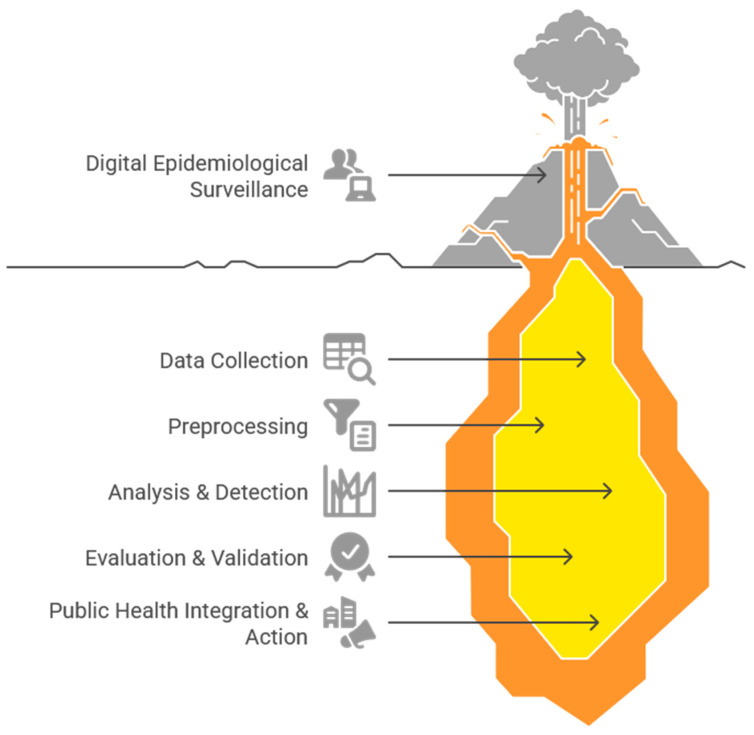
Conceptual framework for digital epidemiological surveillance. Note: This figure was generated using Napkin.

The diverse findings of this review can be best understood when organized into a conceptual framework that illustrates the complete pipeline of digital epidemiological surveillance, from data collection to public health application (see Figure 9). This framework helps structure the remarkable methodological diversity observed in the literature, which ranges from comparative empirical analyses to advanced research based on machine learning. It also accounts for the clear evolution toward increasingly sophisticated approaches over a long period, from pioneering research on dengue in 2003 to recent analyses on emerging threats like COVID-19.

By conceptualizing digital surveillance as a modern extension of traditional syndromic surveillance operating within the field of infodemiology, we can effectively analyze how these methods have been adapted across various geographical contexts and pathogens. The following discussion will therefore analyze our main findings through the key stages of this framework: (1) Data Collection from Digital Sources, (2) Analysis and Performance Evaluation, and (3) Integration with Public Health Systems.

#### 4.1.1. Data Sources and Collection: The Core of Digital Syndromic Surveillance

The first stage of the framework involves gathering data from a diverse digital ecosystem, where search engines and social media are the dominant sources. A recurrent characteristic is the use of search engine data, primarily from Google Trends, often combined with social media platforms like X. These sources function as proxies for health-seeking behaviors and symptom self-reporting, forming the basis of a digital approach to syndromic surveillance. For example, studies have monitored search queries for terms like “pneumonia” or analyzed “sick posts” on platforms like Weibo to detect early signals. The high volume of search queries allows for the detection of broad trends with high correlation to official data, while the unstructured nature of social media provides richer, though often noisier, contextual data that enables deeper analysis like sentiment classification. The adaptability of these methods has been validated across a wide range of pathogens, including influenza, COVID-19, Zika, MERS, and dengue.

#### 4.1.2. Analysis and Performance: From Raw Data to Actionable Signals

Once collected, the raw digital data undergo significant analysis to transform it into actionable public health signals. This review reveals a clear evolution toward increasingly sophisticated analytical approaches, incorporating innovations such as transfer learning, Bayesian analysis, and time series modeling. For instance, studies have leveraged transfer learning to adapt models to new contexts, used Bayesian methods to identify change points in time series, and employed robust ARIMA models for forecasting. These modern techniques facilitate a deeper understanding of the temporal and causal relationships between digital signals and official data.

One of the most notable findings is the ability of these methods to detect trends far ahead of traditional systems. Studies indicate a significant time advantage, with alerts sometimes preceding official notifications by more than a month, as seen in the work of Feldman et al. [42] who reported an average lead time of 43 days. This predictive accuracy is consistently validated through high correlation coefficients (e.g., r > 0.9 in multiple influenza studies) and significant reductions in predictive errors, with low RMSE and MAE values being a common metric of success. However, performance is not uniform; precision and lead time depend heavily on the data source, the analytical method employed, and the specific characteristics of the disease and region.

#### 4.1.3. Public Health Integration and Challenges

The final stage of the framework is the integration of these digital insights with traditional public health systems. A key finding is that nearly all reviewed studies validate their digital data against official reports from recognized institutions like the CDC, WHO, or national health ministries, reinforcing that these tools currently serve as a complement to, not a replacement for, conventional surveillance.

However, this integration faces significant challenges rooted in the field of infodemiology, particularly regarding data quality and representativeness. Several studies explicitly recognize the distorting effects of media noise and panic. For instance, major public announcements or sensationalized news can trigger waves of searches from the “worried well”, creating massive signal spikes that are unrelated to actual case counts. This challenge is increasingly amplified by modern artificial intelligence, where recommendation algorithms can create filter bubbles or “echo chambers”, and generative AI can produce convincing fake news that triggers artificial spikes in digital data, making it difficult to separate from genuine public health events [52,53,54].

Furthermore, potential biases arising from the digital divide can affect the reliability and equity of these systems. This means that populations with lower internet access or different online behaviors, often rural, elderly, or low-income communities, are systematically underrepresented in the data. Consequently, a model trained on this biased data may fail to detect localized outbreaks in these vulnerable groups, compromising health equity, a limitation noted in studies analyzing regions with variable internet penetration. Addressing these challenges is crucial for moving digital surveillance from a set of promising research tools to a fully integrated and reliable component of modern public health response [16,55].

### 4.2. Comparison with Previous Literature

The findings of this review concur with previous research highlighting the utility of digital surveillance. However, to provide a more actionable analysis for researchers and policymakers, the approaches identified in the literature can be classified according to their primary use-case and the resource setting in which they are applied, as summarized in Table 6.

A key distinction emerges between real-time monitoring tools and retrospective models. Real-time “nowcasting” approaches often leverage live data streams from platforms like X to provide immediate situational awareness, which is invaluable during fast-moving outbreaks like influenza or cholera. In contrast, retrospective models frequently use historical data archives, such as Google Trends, to analyze the dynamics of past epidemics (e.g., Zika, Dengue) or to build and validate predictive forecasting models.

Furthermore, the choice of methodology is heavily influenced by the resource context. Studies conducted in high-resource settings often demonstrate the capacity to integrate multiple, complex data streams, including social media, search queries, and even electronic health records, using sophisticated machine learning models. Conversely, research in low-resource settings provides valuable insights into how to maximize the utility of single, highly accessible data sources like Google Trends, often employing more straightforward but effective statistical models.

Beyond classifying the primary studies included in our review, it is also useful to position our work within the context of other recent syntheses in the field. Table 7 provides a direct comparison with several key reviews and pioneering studies, highlighting our unique contribution in terms of methodological scope and practical application.

For example, this study (2025) evaluates the use of social media and digital sources for the early detection of infectious disease outbreaks using retrospective and predictive analyses, machine learning, correlations, and time series analysis from X, Google Trends, health forums, news databases, and epidemiological records. It demonstrates early outbreak detection several weeks in advance with high correlations to official reports; however, it also notes variability in data representativeness depending on the region and digital access.

In contrast, Al-Kenane et al. (2024) [56] examine the relationship between Google Trends and governmental response in Kuwait using time series analysis, Pearson correlations, and bootstrap techniques. They report a high correlation (R ~ 0.71) and note anticipatory changes in policies but with a limited geographical scope. Other studies, such as Melo et al. (2024) [57], Peng Jia et al. (2023) [58], Zhao et al. (2021) [18], and Salathé et al. (2012) [59], are also discussed in relation to their objectives, methodologies, and findings, highlighting innovative approaches, challenges in generalization, and the evolution of digital epidemiology.

Moreover, Melo et al. (2024) [57] share objectives similar to this study, as they also evaluate multiple digital tools, such as Google Trends, X, and mobile applications, in arbovirus surveillance. Their comparative review, based on statistical methods such as ANOVA and correlations, corroborates the advantages identified in early detection and predictive precision. However, Melo et al. [57] also emphasize high methodological variability among studies, a challenge echoed in the current research, stressing the need for greater standardization in the field.

The work by Peng Jia et al. (2023) [58] provides a complementary perspective by focusing on advanced technological innovations such as artificial intelligence, GIS, and digital twins, demonstrating significant improvements in accuracy and real-time detection. Although it does not concentrate exclusively on arboviruses, this study broadens the context of digital surveillance, highlighting the role of smart devices and technological evolution in enhancing epidemiological response. Nonetheless, it acknowledges methodological heterogeneity as a persistent barrier, similar to our findings.

In addition, Zhao et al. (2021) [18] contribute a critical and complementary dimension by analyzing ethical aspects related to digital surveillance in public health. Their approach diverges from operational considerations to address crucial issues such as privacy and civil rights protection, factors essential for ensuring the social acceptance and long-term sustainability of these systems. Although their analysis does not include concrete operational metrics, it emphasizes the importance of balancing technical efficiency with ethical responsibility, a consideration indirectly recognized in our study through the impact of media and digital context.

The pioneering work by Salathé et al. (2012) [59] laid the conceptual foundations of digital epidemiology by highlighting the initial potential of social media and Big Data to reduce outbreak detection times. Even though it lacks the detailed metrics provided by more recent studies, its foundational contribution helps explain how the field has evolved toward more rigorous and quantitative methodological approaches, as exemplified in the present study.

When comparing the current study with that of Shakeri et al. (2021) [60], broad methodological and temporal diversity in the analyzed approaches is evident, ranging from early research on diseases such as dengue to recent studies focused on COVID-19. Notably, there is an increasing use of advanced techniques such as transfer learning, Bayesian analysis, and time series models, especially in the context of respiratory and viral diseases. Moreover, the importance of adapting these methodologies to specific regional contexts and effectively integrating digital sources (such as Google Trends and social networks) with traditional epidemiological surveillance systems is underscored.

The scoping review by Shakeri et al. (2021) [60] provides a broader and more detailed perspective on the use of digital platforms in public health surveillance, covering not only infectious diseases but also areas such as mental health and chronic conditions. This study also highlights significant limitations, such as methodological biases arising from keyword selection and the limited practical application of the results in concrete public health actions. Both studies agree on the need for continuous methodological improvement and stronger integration of digital data with traditional systems, which is vital to maximize the impact of public health interventions. In this regard, it is important to emphasize that the complementarity between these analytical approaches and traditional surveillance enhances the response capacity to health emergencies. This implies not only technological advancement but also the consideration of sociocultural and operational factors that facilitate greater effectiveness in public health interventions derived from digital surveillance.

To provide a more actionable analysis for researchers and policymakers, it is useful to compare the analytical techniques identified in this review. As summarized in Table 8, the choice of method depends on the specific research question, data characteristics, and the desired balance between interpretability and predictive power. Simpler methods like correlation and linear regression offer transparency and are ideal for initial validation, confirming whether a digital data source shows a basic relationship with official case counts. In contrast, more sophisticated approaches like supervised ML are better suited for integrating complex, multi-source data streams to achieve higher predictive accuracy, though often at the cost of interpretability.

Each technique serves a distinct purpose within the digital surveillance toolkit. While time series models like ARIMA are specialized for forecasting diseases with clear seasonal patterns, they may struggle with the unpredictability of novel outbreaks. This is where supervised ML excels, offering the flexibility to model complex interactions from heterogeneous inputs like search queries, mobility data, and social media. NLP is indispensable for unlocking insights from unstructured text, allowing for real-time sentiment analysis and symptom mining from platforms like X. Finally, Bayesian methods offer a crucial advantage by quantifying the uncertainty in predictions, which provides a probabilistic framework to support robust public health decision-making, such as identifying the precise onset of an outbreak.

In summary, by explicitly comparing our approach with that of other studies, it is evident that:

- Methodological Novelty: Our study integrates multiple digital sources and applies advanced machine learning techniques and time series modeling, thereby surpassing the limited scope of research focused on specific geographical or technological contexts.
- Practical Application: Systematic validation against official data and the consideration of media impact and demographic variables reinforce the operational utility of our approach, enabling early outbreak alerts with advantages of up to several weeks.
- Contextualization of Limitations: Whereas previous studies point out limitations in generalization and methodological heterogeneity, our work links these shortcomings with previous empirical evidence and proposes specific strategies (such as improved keyword selection and multi-source integration) to overcome them in future research.

This explicit comparison highlights how our approach contributes to the evolution of digital public health surveillance by offering a more comprehensive methodology that is adaptable to diverse epidemiological and operational contexts.

### 4.3. Methodological Considerations

The analysis of the methods and techniques used in digital surveillance reveals a shared methodological flow, spanning data collection to evaluation, and emphasizes how the incorporation of advanced techniques substantially improves early outbreak detection. In the initial phase, data are collected from diverse digital sources, such as search engines, social networks, specialized platforms, and mobility data, followed by rigorous preprocessing, including normalization and smoothing, to ensure clarity and high-quality epidemiological signals.

#### 4.3.1. Discussion of Specific Techniques

One of the most innovative methodological aspects is the use of transfer learning and Bayesian analysis. For example, some studies (such as Lampos et al., 2021 [28], described in Table 3) have implemented unsupervised models combined with transfer learning techniques. These methods allow for leveraging information previously learned from large volumes of data, thereby facilitating the detection of subtle patterns in new contexts and enhancing predictive capacity in scenarios with limited data. Similarly, the use of Bayesian algorithms, as employed by authors such as Sharpe et al. (2016) [43] and Yan et al. (2017) [34], has proven effective in identifying change points in time series. These algorithms allow for more precise modeling of uncertainty and have, on occasion, resulted in improved correlation with official data, translating into more reliable early alerts.

#### 4.3.2. Clarity in the Integration Process

The integration of digital data with official sources is another fundamental pillar that boosts the predictive accuracy of digital surveillance. For example, the study by Timpka et al. (2014) [29] combined signals from Google Flu Trends with clinical and laboratory data, achieving correlation coefficients as high as 0.96, which demonstrates the synergy between both types of data. This integration not only reinforces the validity of predictive models but also mitigates the biases inherent in relying solely on digital data. An additional example is found in the work by Yousefinaghani et al. (2021) [26], where the simultaneous use of the X API and Google Trends contrasted with official reports (such as those from Johns Hopkins COVID-19) and resulted in a notable improvement in prediction accuracy, with reduced error metrics (RMSE and MAE) and high correlation coefficients (above 75%).

#### 4.3.3. Keyword Selection and Management of Media Impact

Similarly, the proper selection of keywords, implemented through methods ranging from manual identification to automated tools such as Google Correlate, is critical for extracting relevant signals and minimizing digital noise. Specific strategies, as described in studies like those by Shakeri Hossein Abad et al. (2021) [60], have enabled the reduction of interference from media overexposure by adjusting predictions based on variations in informational attention, while ensuring the representativeness of the signals. This approach contributes to a better interpretation of the data, ultimately optimizing early detection and operational response in public health.

The combination of advanced techniques (such as transfer learning and Bayesian analysis) with a rigorous process that integrates digital data with official sources translates into significant improvements in outbreak prediction. The concrete examples provided in Table 3 support the notion that these strategies not only increase early alert capacity (with advantages ranging from several days to weeks) but also reduce predictive errors, positioning these methods as essential complementary tools in epidemiological surveillance.

### 4.4. Study Limitations

Despite the significant advances in digital surveillance presented by the analyzed studies, important methodological limitations deserve a more in-depth discussion. First, there is notable heterogeneity in spatial resolution and temporal granularity, ranging from very local scales (such as municipal level) to global analyses. This variability considerably complicates the comparability and generalization of results, as epidemiological signals may exhibit different patterns depending on the geographic and temporal context. Therefore, it would be necessary to establish minimum standards that allow for more robust comparative analyses, thereby facilitating the interpretation of results across diverse studies [13,60,61].

Another critical point is the selection of keywords, a fundamental procedure for ensuring the quality and representativeness of digital signals. Currently, many studies rely on manual or semi-automated methodologies for determining these keywords, which may not fully capture the complexity and evolution of digital language, especially in heterogeneous cultural or linguistic contexts. This could result in biases in the early identification of outbreaks, potentially underestimating or overestimating certain terms based on subjective criteria or technical limitations. Therefore, moving toward more sophisticated methods, such as machine learning-based language models, could allow for a more dynamic, adaptive, and precise selection of key terms [42,60,62].

Another substantial challenge lies in the insufficient consideration of demographic and technological variables in most studies. The lack of detailed analyses regarding the demographic composition of digital users, including factors such as age, gender, education, socioeconomic status, and location, can limit the representativeness of the obtained epidemiological signals. Moreover, the digital divide, marked by disparities in access to and use of technology between urban and rural areas or between countries with different socioeconomic levels, can significantly bias the results, hindering their generalization and universal applicability. Thus, integrating detailed demographic analyses and studies on technological penetration would allow for a better interpretation of the data and tailored conclusions for specific realities [63,64].

Although most research validates its findings through comparison with official data, the systematic integration of these digital methodologies with traditional epidemiological surveillance systems remains limited. There is still a need to develop clear and standardized protocols that facilitate the effective and transparent combination of both data sources. In this regard, internationally coordinated multicenter studies could provide the necessary foundation to establish consensus standards that ensure external validity and allow for reliable and generalized application of the results on a global scale [16,60].

### 4.5. Clinical Implications and Recommendations for Future Research

Digital surveillance presents itself as a promising complement to traditional epidemiological surveillance systems by enabling the early detection of outbreaks and near real-time analysis of epidemiological trends. However, to fully exploit this potential and overcome the current challenges, it is necessary to implement standardized protocols and more robust integration strategies, combined with an interdisciplinary approach that brings together technical experts, epidemiologists, and social scientists [1,65].

#### 4.5.1. Clear and Actionable Recommendations

Standardization of Protocols: It is imperative to develop multicenter studies that use uniform methods in all phases of the analysis. This would include the adoption of standardized protocols for keyword selection, whether through manual or automated methods, and for data preprocessing (normalization, smoothing, filtering). For example, common guidelines could be established that integrate similar validation metrics to those employed in the works of Brancato et al. (2024) [66] or Jacobson et al. (2024) [67], thus facilitating the comparison of results across different studies and geographical contexts.

Systematic Integration of Demographic Analysis: It is recommended to systematically incorporate geographical, socioeconomic, and demographic variables into predictive models. This approach would improve the representativeness and accuracy of epidemiological signals, reducing biases arising from regional variations in access to and use of digital technologies. To achieve this, future research should explore advanced statistical techniques such as model weighting or post-stratification, where digital data are adjusted against census data to create more nationally representative samples. Furthermore, presenting results stratified by geographic or demographic groups, rather than as a single aggregate figure, would provide more granular and equitable public health insights [60,64].

- Use of Emerging Technologies: The adoption of artificial intelligence tools and advanced techniques, such as metagenomic analysis, could optimize outbreak detection. It is recommended to implement automated classification and filtering techniques to analyze large volumes of data, thereby increasing the sensitivity and predictive capacity of digital systems, particularly when combined with traditional surveillance sources [68].
- Mitigation of Media-Driven Noise: To counteract the effects of the infodemic, models should be designed to distinguish between general “chatter” and true symptom-related signals. This could involve multi-stream analysis that compares symptom searches against news trends or the integration of data sources less susceptible to media influence, such as participatory surveillance systems.
- Longitudinal Evaluations: It is suggested to conduct long-term follow-up studies that evaluate the efficacy, stability, and cost-effectiveness of digital systems in various epidemiological contexts. This approach would not only provide robust evidence on the sustainability of digital surveillance but also help refine and improve predictive models over time.

#### 4.5.2. Interdisciplinary Perspective

To address the complexity of digital analysis in public health, it is essential to foster collaboration among technical experts, epidemiologists, and social scientists. Such interdisciplinary collaboration would allow for the following:

- Integrating Technical and Contextual Knowledge: Technology specialists can optimize algorithms and predictive models, while epidemiologists contribute their understanding of disease dynamics and social scientists provide insights into cultural, demographic, and behavioral factors that are essential for interpreting digital signals.
- Designing Adapted and Equitable Interventions: This collaborative approach will facilitate the design of public health interventions that are both precise and adapted to local realities, maximizing the impact on outbreak prevention and control.
- Developing Holistic Solutions: By combining skills and knowledge from various disciplines, it is possible to develop comprehensive solutions that address both operational and ethical aspects, ensuring that digital surveillance is implemented responsibly and with high standards of effectiveness.

Although current evidence widely supports the predictive capacity of digital tools in epidemiological surveillance, methodological and operational challenges persist that hinder their clinical generalization. Therefore, it is recommended to adopt an interdisciplinary and collaborative approach that combines advanced technological innovations with rigorous methodological designs, thereby strengthening the public health system’s response capacity in the face of epidemiological emergencies.

### 4.6. From Prediction to Action: Early Warning Mechanisms and Their Impact

While the title of this review emphasizes “early warning”, a crucial distinction must be made between a model’s predictive accuracy and its function as a true warning mechanism. The value of digital surveillance lies not just in forecasting trends, but in its ability to translate those forecasts into timely, actionable alerts for public health officials. Our review identified three primary mechanisms through which the included studies operationalize these warnings.

The first and most straightforward mechanism is based on anomaly detection. These systems function by establishing a baseline of normal digital activity and triggering an alert when data deviates significantly from this pattern. For example, studies have monitored search volumes for terms like “pneumonia” and issued a warning upon detecting an abnormal spike, suggesting a potential outbreak before official case counts rise [5,44]. This approach is effective for capturing sudden changes but can be sensitive to media-driven noise.

A second, more structured mechanism relies on pre-defined thresholds. These approaches often adapt established epidemiological methods, such as the Moving Epidemic Method, to digital data streams. A warning is issued only when the signal (e.g., search interest for RSV) crosses a statistically defined intensity threshold, providing a more robust and less arbitrary alert system [30,47].

The third mechanism involves supervised classification. Here, machine learning models are trained to identify and categorize specific infection-related content, such as tweets describing symptoms or social media posts from individuals who self-identify as ill. An early warning is then triggered when the volume or proportion of this classified content surpasses a certain frequency, effectively creating a real-time cohort of “digital cases” [41,49,50].

However, a critical finding of this review, and a key limitation of the current field, is the profound gap between demonstrating a model’s predictive accuracy and evaluating its real-world impact on public health response. While nearly all studies validate their models against official case data, we found a scarcity of research that measures whether the “early warnings” generated by these digital systems led to concrete actions (e.g., increased local testing, targeted public health messaging, resource allocation) or ultimately altered the trajectory of an outbreak. This evidence gap highlights the critical next step for the field: moving beyond prediction and toward a formal evaluation of translational impact.

## 5. Conclusions

The evidence gathered in this review demonstrates that the integration of diverse digital sources, such as search engines, social networks, and specialized databases, enables the anticipation of infectious disease outbreaks with a considerable time advantage over traditional surveillance systems. These methods, which employ advanced statistical analysis and machine learning techniques, consistently achieve high correlation and precision when compared with official data.

Nonetheless, challenges persist due to heterogeneity in spatial and temporal resolution, inconsistencies in keyword selection and processing, varying strategies for managing media noise, and the limited incorporation of demographic and behavioral data. These factors contribute to bias and limit the clinical generalizability and external validity of current approaches.

To address these limitations, actionable steps must be taken. First, methodological protocols should be standardized across studies, particularly in the selection and preprocessing of keywords. This could involve the use of NLP-based taxonomies, crowd-sourced dictionaries, or biomedical ontologies to ensure consistency and contextual relevance. Second, integrating demographic, geographic, and behavioral dimensions into models can help mitigate bias and improve representativeness. Finally, leveraging artificial intelligence for dynamic adaptation to emerging trends and data patterns can further enhance system accuracy. Together, these strategies will facilitate real-time, informed decision-making and lay the groundwork for more robust, equitable, and effective digital public health surveillance systems in future epidemiological emergencies.

## Figures and Tables

**Table 1:** General characteristics of the included studies.

Author and Year	Study Design	Data Collection Period	Location	Type of Disease or Outbreak
Dai et al. (2020) [5]	Quantitative comparative empirical	2015–2020	China	COVID-19
Lampos et al. (2010) [31]	Quantitative empirical	2009	United Kingdom	Influenza (H1N1)
Lampos et al. (2021) [28]	Observational/modeling	2011–2020	USA, United Kingdom, Australia, etc.	COVID-19
Van de Belt et al. (2018) [30]	Comparative exploratory	2015–2017	Netherlands	MRSA
Timpka et al. (2014) [29]	Open cohort	2007–2012	Sweden	Influenza
McGough et al. (2017) [32]	Retrospective multivariable forecasting	2015–2016	Latin America	Zika
Yousefinaghani et al. (2021) [26]	Observational, retrospective, and predictive	2020	USA and Canada	COVID-19
Wittwer et al. (2023) [33]	Cross-sectional comparative	2020	Brazil	COVID-19
Shin et al. (2016) [8]	Observational correlational	2015	South Korea	MERS
Yan et al. (2017) [34]	Systematic review	2006–2016	International	Various
Strauss et al. (2017) [35]	Observational correlational	2004–2014	Venezuela	Dengue
Chunara et al. (2012) [36]	Observational	2011	Haiti	Cholera
Barboza et al. (2014) [37]	Quantitative evaluation	2010	International	Various
Kogan et al. (2021) [6]	Early warning	2020	USA	COVID-19
Verma et al. (2018) [38]	Cross-sectional correlational	2016	India	Dengue, malaria, etc.
Santillana et al. (2015) [39]	Machine learning	2013–2015	USA	Influenza (ILI)
Majumder et al. (2016) [40]	Retrospective	2015–2016	Colombia	Zika
Samaras et al. (2020) [10]	Comparative	Influenza season	Greece	Influenza
Li et al. (2021) [41]	Retrospective	2020	USA	COVID-19
Feldman et al. (2019) [42]	Database development	10 months	Global	114 diseases
Sharpe et al. (2016) [43]	Retrospective comparative	2012–2015	USA	Influenza (ILI)
Porcu et al. (2023) [44]	Retrospective	2020–2021	Italy	COVID-19
Lu et al. (2018) [45]	Retrospective observational	2012–2016	Boston, USA	Influenza
Chan et al. (2011) [46]	Real-time monitoring	2003–2010	Bolivia, Brazil, India, etc.	Dengue
Wang et al. (2023) [47]	Outbreak prediction	5 years	Japan, Germany, Belgium	RSV
Alessa and Faezipour (2019) [48]	Retrospective observational	2018	USA (Connecticut)	Influenza
Broniatowski et al. (2013) [49]	Observational infoveillance	2012–2013	USA (National and NYC)	Influenza
Shen et al. (2020) [50]	Retrospective observational	2019–2020	China	COVID-19
Broniatowski et al. (2015) [51]	Retrospective observational study	20 November 2011—16 March 2014	Baltimore, Maryland, USA (inner-city hospital)	Influenza

COVID-19: coronavirus disease 2019; H1N1: a subtype of the influenza A virus (H1N1); MRSA: methicillin-resistant Staphylococcus aureus; MERS: Middle East respiratory syndrome; ILI: influenza-like illness.

**Table 2:** Digital data sources and platforms employed.

Author and Year	Specific Digital Platforms and Tools	Integration with Traditional Surveillance Systems
Dai et al. (2020) [5]	Baidu Search Engine	Comparison with the traditional case reporting system
Lampos et al. (2010) [31]	X	Calibration of the “flu-score” with HPA data
Lampos et al. (2021) [28]	Google Search and news data	Comparison with official case and death data
Van de Belt et al. (2018) [30]	Coosto (social media monitoring) and Google Trends	Comparison with official notifications in the SO ZI/AMR system
Timpka et al. (2014) [29]	Google Flu Trends, Healthcare Direct/1177, Google Analytics	Comparison with clinical and laboratory data on influenza
McGough et al. (2017) [32]	Google Search, X, HealthMap	Integration with Zika data reported by PAHO and health ministries
Yousefinaghani et al. (2021) [26]	X API and Google Trends	Comparison with official data (Johns Hopkins COVID-19)
Wittwer et al. (2023) [33]	Brazil Sem Corona and GitHub data	Integration with PS and TS data to improve prediction
Shin et al. (2016) [8]	Google Trends, Topsy	Comparison with official MERS data
Yan et al. (2017) [34]	Google Flu Trends, Google Trends, Baidu, X, ProMED-mail, HealthMap	Discussion on complementarity with traditional systems
Strauss et al. (2017) [35]	Google Dengue Trends	Comparison and proposal for complementarity with the surveillance system
Chunara et al. (2012) [36]	HealthMap and X	Comparison with official data from the MSPP
Barboza et al. (2014) [37]	Argus, BioCaster, GPHIN, HealthMap, MedISys, ProMED-mail	Comparative evaluation with official BHI data
Kogan et al. (2021) [6]	Google Trends, X, UpToDate, GLEAM, Apple Mobility, Cuebiq, Kinsa Thermometer	Integration of digital proxies with cases, deaths, and ILI
Verma et al. (2018) [38]	Google Trends and Google Correlate	Comparison with the IDSP surveillance system
Santillana et al. (2015) [39]	Google Trends, X, athenahealth, FluNearYou	Comparison of predictions with CDC reports
Majumder et al. (2016) [40]	HealthMap and Google Trends	Validation with official INS data
Samaras et al. (2020) [10]	Google Trends, X API (Tweepy and Pytrends)	Comparison with official influenza data in Europe
Li et al. (2021) [41]	X Standard Search API	Comparison with official systems based on searches and news
Feldman et al. (2019) [42]	GDELT Global Knowledge Graph and Google Translate API	Comparison with WHO (DON) reports
Sharpe et al. (2016) [43]	Google Flu Trends, HealthTweets, Wikipedia	Comparison with CDC official reports
Porcu et al. (2023) [44]	Google Trends	Validation with RT-PCR data
Lu et al. (2018) [45]	Google Trends, X, athenahealth, Flu Near You	Validation with data from the Boston Public Health Commission
Chan et al. (2011) [46]	Google Search queries	Comparison with data from ministries of health and WHO
Wang et al. (2023) [47]	Google Trends	Complement for clinical surveillance
Alessa and Faezipour (2019) [48]	X	Validation with CDC and hospital data
Broniatowski et al. (2013) [49]	X API (HealthTweets and Google Flu Trends)	Validation with CDC and NYC Department of Health reports
Shen et al. (2020) [50]	Weibo	Comparison with official data from the China CDC
Broniatowski et al. (2015) [51]	X (HealthTweets)	Comparison with hospital data (laboratory cases and ILI in ED)

CDC: Centers for Disease Control and Prevention; HPA: Health Protection Agency; PAHO: Pan American Health Organization; WHO: World Health Organization; API: application programming interface; GPHIN: Global Public Health Intelligence Network; RT-PCR: reverse transcription-polymerase chain reaction; NPIs: non-pharmaceutical interventions; DON: Disease Outbreak News.

**Table 3:** Methods for data analysis and processing.

Author and Year	Comparison Method	Detection Method	Preprocessing	Analytical Techniques
Dai et al. (2020) [5]	Correlation analysis between anomalous peaks and official reports	Abnormal increase in ILI and searches (e.g., “pneumonia”, “SARS”)	Smoothing (7-day moving average)	ANOVA, linear regression, correlation
Lampos et al. (2010) [31]	Comparison of “flu-score” in tweets versus ILI rates	Calculation of “flu-score” from tweets	Stop word removal, stemming, smoothing	Linear regression, LASSO, supervised learning
Lampos et al. (2021) [28]	Comparison of online queries with official COVID-19 data	Unsupervised models and transfer learning with symptoms	Normalization and weighting of symptoms	Elastic net, Gaussian processes, correlation
Van de Belt et al. (2018) [30]	Comparison of outbreaks detected on social networks with official reports	Detection on social media and Google Trends	Thresholds in social media and Google Trends	Descriptive statistics, ROC analysis, correlation
Timpka et al. (2014) [29]	Comparison of eHealth data with clinical and laboratory cases	Correlation of eHealth data with clinical data	Weekly adjustment, detrending	Linear regression, autoregressive models, correlation
McGough et al. (2017) [32]	Predictive models of Zika cases with digital data	Case prediction using digital signals	Log transformations and normalization	Elastic net, cross-validation, autoregressive models
Yousefinaghani et al. (2021) [26]	Comparison of digital time series with COVID-19 cases	Anomaly analysis in tweets and searches	Keyword filtering and geolocation	Anomaly analysis, regression, validation
Wittwer et al. (2023) [33]	Comparison of self-reported infection rates with official data	Estimation of infection rates from self-reports	LOESS smoothing of fluctuations	Autoregressive models, AIC, variable combination
Shin et al. (2016) [8]	Correlation between digital data and official cases	Lag correlation between digital data and cases	Normalization and word selection	Spearman and lag analysis
Yan et al. (2017) [34]	Correlation analysis and detection of digital signals	Detection of digital signals in official reports	Categorization and noise elimination	Correlation, Bayesian algorithms, signal detection
Strauss et al. (2017) [35]	Comparison of digital surveillance with reported dengue cases	Digital surveillance based on dengue searches	Normalization and volume conversion	Linear regression, correlation analysis
Chunara et al. (2012) [36]	Correlation analysis between tweets and cholera reports	Analysis of HealthMap and X reports	Filtering and selection of key terms	Exponential fit, Euler–Lotka equation
Barboza et al. (2014) [37]	Evaluation of biosurveillance with media signals	Media searches validated by human assessment	Manual filtering and duplicate removal	Poisson regression, rate calculations
Kogan et al. (2021) [6]	Comparison of digital proxies with case and death data	Modeling digital proxies and official data	Smoothing and scaling of digital proxies	Exponential growth, harmonic mean, correlation
Verma et al. (2018) [38]	Correlation between search patterns and outbreaks in India	Identification of terms in Google Correlate	Selection of terms in Google Correlate	Correlation analysis and time series analysis
Santillana et al. (2015) [39]	Prediction of ILI by combining multiple digital sources	Prediction of ILI activity using multiple proxies	Normalization and mapping of digital sources	LASSO regression, SVM, AdaBoost
Majumder et al. (2016) [40]	Estimation of Zika transmission using digital data	Modeling Zika transmission with IDEA	Scaling and smoothing of Google Trends	Non-linear optimization, SSD minimization
Samaras et al. (2020) [10]	Predictive modeling of influenza with ARIMA	Epidemic activity prediction using ARIMA	Elimination of duplicates in X	ARIMA(X) models, predictive analysis
Li et al. (2021) [41]	Classification of COVID-19 tweets and lead time analysis	Classification of tweets as COVID-19 alerts	Tokenization and lemmatization of tweets	Supervised classification and sentiment analysis
Feldman et al. (2019) [42]	Validation of outbreak detection with WHO reports	Outbreak detection in news articles	Automatic translation and tag-based filtering	Naïve Bayes, SVM, bidirectional LSTM
Sharpe et al. (2016) [43]	Detection of changes in time series using Bayesian methods	Identification of change points in time series	Normalization and weekly grouping	Bayesian change point models
Porcu et al. (2023) [44]	Detection of outliers in searches using ARMA and EWMA	Detection of epidemic signals in searches	Scaling adjustment from 0 to 100	ARMA, EWMA, outlier detection
Lu et al. (2018) [45]	Comparison of ARGO models vs. simple autoregressives	Prediction of influenza with ensemble models	Filtering out irrelevant terms	Multivariable regression and ensemble methods
Chan et al. (2011) [46]	Fitting linear models to dengue searches	Univariate linear regression with dengue searches	Replacement of spurious peaks	Univariate linear regression
Wang et al. (2023) [47]	Correlation between Google Trends and clinical surveillance	Definition of thresholds with the Moving Epidemic Method	Exclusion of atypical years (2020–2021)	Moving Epidemic Method (MEM)
Alessa and Faezipour (2019) [48]	Classification of tweets with FastText and linear regression	Regression and classification of tweets	Stemming and stop word removal	FastText and linear regression
Broniatowski et al. (2013) [49]	Tweet filtering for influenza detection	Supervised classification of influenza tweets	Filtering in tweet stages	SVM, logistic regression
Shen et al. (2020) [50]	Granger causality analysis between “sick posts” and case counts	Case prediction using Granger causality and supervised models	Classification into “sick” versus others	Random forest classifier and OLS regression
Broniatowski et al. (2015) [51]	Estimation of influenza prevalence using tweets and counts	Estimation of ILI prevalence from X	Normalization of tweet volumes	ARIMAX analysis and logistic regression

ANOVA: analysis of variance; LASSO: least absolute shrinkage and selection operator; LOESS: locally estimated scatterplot smoothing; ROC: receiver operating characteristic; AIC: Akaike information criterion; SSD: sum of squared differences; ARIMA: autoregressive integrated moving average; ARIMAX: ARIMA with exogenous variables; SVM: support vector machine; OLS: ordinary least squares; MAE: mean absolute error; MAPE: mean absolute percentage error; AUC: area under the curve.

**Table 4:** Performance in early detection and precision.

Author and Year	Lead Time	Detection Rate	Precision
Dai et al. (2020) [5]	20 days before the official alert	High correlations; no specific rate reported	High correlation coefficients
Lampos et al. (2010) [31]	Tweets within hours; HPA takes 1–2 weeks	Correlations 81.78–85.56%	Cross-validation ~89–94%
Lampos et al. (2021) [28]	Cases: 16.7 days before, deaths: 22.1 days before	Correlation r ≈ 0.82–0.85	Evaluated with AUC and MAE
van de Belt et al. (2018) [30]	Outbreaks detected 1–2 days earlier	Sensitivity 20%, specificity 96%	AUC, sensitivity, specificity
Timpka et al. (2014) [29]	GFT 2 weeks earlier; telenursing varies	GFT r = 0.96, telenursing r ≈ 0.95–0.97	Pearson r, RMSE
McGough et al. (2017) [32]	Forecasts 1–3 weeks earlier	Measured by predictive error (rRMSE)	RMSE, rRMSE, Pearson ρ
Yousefinaghani et al. (2021) [26]	83% of waves detected 1 week early	100% of symptoms detected in US Category I	RMSE, MAE, correlations > 75%
Wittwer et al. (2023) [33]	Lead time depends on participation	High correlation in cities with good participation	RMSE, MAE, Pearson correlation
Shin et al. (2016) [8]	3–4 days prior to confirmation	Correlations > 0.7, up to 0.9	Significant correlations (*p* < 0.05)
Yan et al. (2017) [34]	1–12 days before official reports	Alerts 1–12 days early, variable correlation	Moderate to high depending on the method
Strauss et al. (2017) [35]	Early alert before update	r = 0.87 during epidemic weeks	R^2^ = 0.75 in regression
Chunara et al. (2012) [36]	Daily updates; official data delayed 1–2 days	ρ ≈ 0.80 during growth phases	Variability in Re (1.54 to 6.89)
Barboza et al. (2014) [37]	Detects events before publication	C-DR 83–95%, I-DR 47–92%	Statistical differences in I-Se
Kogan et al. (2021) [6]	Case increases 2–3 weeks earlier	Combined sensitivity up to 0.75	Precision 0.90–0.98 in proxies
Verma et al. (2018) [38]	Google Trends anticipates 2–3 weeks	r > 0.80 for chikungunya and dengue	Chikungunya r = 0.82–0.87
Santillana et al. (2015) [39]	Prediction up to 4 weeks before	Real-time prediction r = 0.989	RMSE 0.176% ILI, reduced MAPE
Majumder et al. (2016) [40]	Near real-time estimates	No detection rate reported; estimation of R0	Good SSD model fit
Samaras et al. (2020) [10]	Searches and tweets anticipate 2–3 weeks	Pearson R ≈ 0.933–0.943	MAPE ≈ 18.7–22.6%
Li et al. (2021) [41]	Detects signals 16 days in advance	Signal strategy identifies alerts	High classification precision
Feldman et al. (2019) [42]	Outbreaks detected on average 43.4 days earlier	94% of outbreaks detected before WHO	Recall 88.8%, precision 86.1%
Sharpe et al. (2016) [43]	Google alerts changes 1–2 weeks earlier	Google: sensitivity 92%, PPV 85%	Google shows the best performance
Porcu et al. (2023) [44]	Epidemics detected 7–8 weeks before	PPV 80% in Lombardy, <50% in Marche	High correlation in areas with high connectivity
Lu et al. (2018) [45]	Nowcasting and forecasting 1 week ahead	Correlations of 0.98 (nowcast) and 0.94 (forecast)	Low RMSE, MAE, and MAPE
Chan et al. (2011) [46]	Real-time available data	Correlations 0.82–0.99	Good correlation fit
Wang et al. (2023) [47]	Almost immediate data	Japan r = 0.87, Germany r = 0.65	Good threshold estimation
Alessa and Faezipour (2019) [48]	Almost real-time	96.29% correlation with CDC	F-measure 89.9%
Broniatowski et al. (2013) [49]	Tweets available up to 2 weeks in advance	National r = 0.93, municipal r = 0.88	Lower MAE in the infection model
Shen et al. (2020) [50]	Predicts cases 14 days earlier	Sick posts explain 12.8% of variance	High standardized coefficients
Broniatowski et al. (2015) [51]	Tweets ahead of official data	High correlation at the municipal level	85% accuracy in trend prediction

PPV: positive predictive value; RMSE: root mean square error; MAE: mean absolute error; MAPE: mean absolute percentage error; rRMSE: relative root mean square error; AUC: area under the curve; R^2^: coefficient of determination; ρ (rho): correlation coefficient; Re: effective reproduction number.

**Table 5:** Complementary characteristics and contextual variables.

Author and Year	Spatial Resolution and Temporal Granularity	Keyword Selection Process	Measurement of Media Impact	Demographic and Usage Characteristics
Dai et al. (2020) [5]	Regional (Wuhan, China); daily and weekly data	Manual selection (“pneumonia”, “SARS”)	Not evaluated	Not specified
Lampos et al. (2010) [31]	Urban centers (10 km radius); daily and weekly aggregation	Manual selection and LASSO	Not directly measured	5.5 million X users (United Kingdom)
Lampos et al. (2021) [28]	National; daily data	19 symptom-based sets	Minimizes panic effect in the autoregressive model	Application in multiple countries, no demographic details
Van de Belt et al. (2018) [30]	Provinces; daily data	Boolean searches in Google Trends	Not explicitly evaluated	Geographic information by province
Timpka et al. (2014) [29]	County; daily data	ICD-10 and grouping in telenursing	Correlation between media coverage and GFT	Age distribution in RIR
McGough et al. (2017) [32]	National; weekly data	LASSO and penalized regression	Not measured; low influence mentioned	Data profiles by country, no demographics
Yousefinaghani et al. (2021) [26]	States/provinces; weekly data	Predefined symptom lists	Indirect impact by comparing preventive term usage	Geolocation of tweets by state/province
Wittwer et al. (2023) [33]	Municipalities; daily data	Questionnaire based on COVID-19 symptoms	Impact of media campaigns on participation	Participation rates and urban differences
Shin et al. (2016) [8]	National; daily data	Basic and extended terms (“MERS”, “hospital”)	Recognizes media noise	Aggregated search and tweet data
Yan et al. (2017) [34]	Local/global; daily to weekly data	Relevance and specificity-based selection	Evaluation of media noise	Lack of detailed user data
Strauss et al. (2017) [35]	National; weekly data	Spanish terms for dengue	Annual variation in searches vs. incidence	Impact of internet penetration
Chunara et al. (2012) [36]	Departments and arrondissements; daily data	Searches for “cholera” and hashtags	Media amplification effect	Geographic and demographic biases
Barboza et al. (2014) [37]	Country-level events; monthly data	Defined by epidemiologists	Comparison of media and official source signals	Language distribution and regional impact
Kogan et al. (2021) [6]	States; daily data	COVID-19-related terms	Analysis of bias in digital proxies	Differences in activity and adherence to NPIs
Verma et al. (2018) [38]	States; weekly data	Terms with high correlation (Google Correlate)	Search explosion preceding the report	Internet penetration in Haryana and Chandigarh
Santillana et al. (2015) [39]	National; weekly data	Terms based on previous studies	Captures media effects in search variation	Aggregated national level, no demographic details
Majumder et al. (2016) [40]	National; aggregated data	Keyword “Zika” in Google Trends	Comparison of curves, not evaluating noise	Aggregated data, no demographic details
Samaras et al. (2020) [10]	National (Greece); aggregated data	Terms in Greek	Media bias in searches and tweets	Limitations in tweet geolocation
Li et al. (2021) [41]	State-level (USA); daily data	Keyword “coronavirus”	Signal ratio as an indicator of public opinion	Filtered by location in the USA
Feldman et al. (2019) [42]	Global; updates every 15 min	Filtering by GDELT and name databases	Lead time of 43.4 days and 94% outbreak coverage	No demographic characteristics; only media data
Sharpe et al. (2016) [43]	National; weekly data	Implicit terms in each source	Evaluation of discrepancies in changes	Aggregated data, no demographic details
Porcu et al. (2023) [44]	Regions (Italy); weekly data	Italian translation of symptoms	Search volume as a proxy for alerts	Variability in internet access by region
Lu et al. (2018) [45]	City; weekly data	Specific terms for Boston	Media influence in method comparison	Emergency room visits (age, gender, ethnicity)
Chan et al. (2011) [46]	National; weekly/monthly data	Selection based on correlation with official data	Not reported	Not reported
Wang et al. (2023) [47]	National and regional; weekly data	Term “RSV” or “RS virus”	Mitigation of media impact with MEM	No specific details reported
Alessa and Faezipour (2019) [48]	State (Connecticut); weekly data	11 verified keywords	Not directly measured	No demographic characteristics detailed
Broniatowski et al. (2013) [49]	Municipal, regional, and national; weekly data	Keyword list and previous models	Sensitive to media “chatter”	Possible biases due to underrepresentation of users
Shen et al. (2020) [50]	National and provincial; daily data	167 keywords per daily observation	Comparison between “sick posts” and other posts	User pool with age and gender composition
Broniatowski et al. (2015) [51]	Municipal (hospital); weekly data	269 health-related terms filtered in stages	Filtering to reduce media “chatter”	Data from pediatric and adult patients

ICD-10: International Classification of Diseases, Tenth Revision; NPIs: non-pharmaceutical interventions.

**Table 6:** Use-Case Classification of Digital Surveillance Approaches.

Use Case/ Purpose	Typical Platforms	Common Diseases	Contextual Considerations (High vs. Low Resources)
Real-Time Monitoring (“Nowcasting”)	X API, participatory systems (FluNearYou), news data (GDELT)	Influenza, COVID-19, Cholera	High resource: Integration of multiple real-time data streams. Low resource: Reliance on free social media platforms.
Retrospective Analysis and Modeling	Google Trends archives, historical social media data	Dengue, Zika, MERS	High and low resource: Accessible in both contexts, as historical data are often freely available.
Predictive Forecasting	Combination of multiple sources (search queries, social media, clinical data)	Influenza, COVID-19, RSV	High resource: Requires high-quality longitudinal data and computational power for complex ML models. Low resource: More challenging; often relies on simpler time series models.

**Table 7:** Comparison of results with other studies.

Authors/Study	Objective/Scope	Methodology/ Techniques	Data Sources	Key Findings	Impact/ Context	Advantages/ Disadvantages
This study (2025)	To evaluate the use of social media and digital sources for early detection of infectious disease outbreaks.	Retrospective and predictive analysis; use of machine learning, correlations, and time series analysis.	X, Google Trends, health forums, news databases, epidemiological records.	Outbreaks anticipated several weeks in advance; high correlation with official reports.	Impact of media context and digital penetration on data quality.	✔ Integration of multiple digital sources; validation with official data. ✘ Variability in data representativeness depending on region and digital access.
Al-Kenane et al. (2024) [56]	Relationship between Google Trends searches and government response in Kuwait.	Time series analysis; Pearson and bootstrap.	Google Trends (English and Arabic).	High correlation (R ~ 0.71); anticipates policy changes.	Incorporates bilingual analysis and effects of government measures.	✔ Innovative and robust approach. ✘ Limited to Kuwait and psychological variables.
Melo et al. (2024) [57]	To evaluate digital tools for arbovirus surveillance and early detection.	Review with comparative analysis; ANOVA and correlations.	Google Trends, X, apps, social media, and official data.	Early detection (days to weeks); high precision in outbreak prediction.	Considers media influence and spatial data resolution.	✔ Comprehensive comparison of tools and contexts. ✘ High variability between studies.
Peng Jia et al. (2023) [58]	Review of technological innovations (AI, GIS, digital twins) in epidemiological surveillance.	Synthesis in Annual Review of Public Health.	Geospatial data, EHRs, big data, electronic reporting.	Improved accuracy, timeliness, and real-time detection.	Impact of smart devices and digital evolution in public health.	✔ Highlights key advances in surveillance. ✘ Study heterogeneity; requires integration with other systems.
Zhao et al. (2021) [18]	Ethical analysis of digital surveillance in infectious diseases.	Systematic review with theoretical focus on privacy and civil rights.	Big data, EHRs, digital surveillance.	Assesses ethical risks vs. benefits; correlation with official reports.	Emphasizes the need to balance surveillance and privacy.	✔ Strong theoretical framework on digital surveillance ethics. ✘ Does not address operational metrics.
Salathé et al. (2012) [59]	Impact of big data and social media on digital epidemiology.	Narrative review and Editors’ Outlook.	Social media, mobile phones, online searches.	Reduced outbreak detection times.	Potential for early alerts vs. technical and bias challenges.	✔ Pioneer in digital epidemiology. ✘ Lacks detailed error metrics.

✔: Advantages; ✘: Disadvantages.

**Table 8:** Comparison of analytical methods for digital epidemiological surveillance: strengths, limitations, and ideal applications.

Method/Technique	Strengths	Limitations	Ideal Use Case and Examples
Correlation and Linear Regression [56,57]	Simple, interpretable—Low computational cost	Assumes linearity—Sensitive to outliers (e.g., media panic spikes)	Initial validation of digital data relevance in disease monitoring.
Time Series Models (e.g., ARIMA) [10,51]	Strong for forecasting—Handles seasonality	Less flexible to sudden changes—Requires data transformation	Short-term forecasts for diseases with seasonal patterns (e.g., flu, RSV).
Supervised ML (e.g., SVM, LASSO, RF) [18,39,58]	Captures complex patterns—Variable selection (e.g., LASSO)	Risk of overfitting—Opaque (“black box”)—Needs large datasets	Integrating diverse sources (searches, mobility, social media) into predictive models.
Natural Language Processing (NLP) [42,57]	Extracts insights from unstructured text—Captures context and nuance	Sensitive to slang/errors—Ambiguity in word meaning	Sentiment and symptom mining from social media for real-time public health signals.
Bayesian Methods [43]	Quantifies uncertainty—Updates with new evidence	Computationally intensive—Sensitive to prior assumptions	Change-point detection in disease trends, e.g., outbreak onset.

Abbreviations: ML: machine learning; SVM: support vector machine; LASSO: least absolute shrinkage and selection operator; RF: random forest; NLP: natural language processing; RSV: respiratory syncytial virus.

## Data Availability

The raw data supporting the conclusions of this article will be made available by the authors on request.

## Abbreviations

The following abbreviations are used in this manuscript:

CDC	Centers for Disease Control and Prevention
HPA	Health Protection Agency
PAHO	Pan American Health Organization
WHO	World Health Organization
API	Application programming interface
GPHIN	Global Public Health Intelligence Network
MEM	Moving epidemic method
RT-PCR	Reverse transcription-polymerase chain reaction
NPIs	Non-pharmaceutical interventions
DON	Disease Outbreak News
ANOVA	Analysis of variance
LASSO	Least absolute shrinkage and selection operator
LOESS	Locally estimated scatterplot smoothing
ROC	Receiver operating characteristic
AIC	Akaike information criterion
SSD	Sum of squared differences
ARIMA	Autoregressive integrated moving average
ARIMA	ARIMA with exogenous variables
SVM	Support vector machine
OLS	Ordinary least squares
RMSE	Root mean square error
MAE	Mean absolute error
MAPE	Mean absolute percentage error
AUC	Area under the curve
R^2^	Coefficient of determination
ρ (rho)	Correlation coefficient
Re	Effective reproduction number
